# How does the media portray drinking water security in Indigenous communities in Canada? An analysis of Canadian newspaper coverage from 2000-2015

**DOI:** 10.1186/s12889-017-4164-4

**Published:** 2017-03-27

**Authors:** Steven Lam, Ashlee Cunsolo, Alexandra Sawatzky, James Ford, Sherilee L. Harper

**Affiliations:** 1 Department of Population Medicine, University of Guelph, 50 Stone Rd E, Guelph, ON N1G 2W1 Canada; 2 Labrador Institute of Memorial University, Happy Valley-Goose Bay, Newfoundland and Labrador Canada; 3 Department of Geography, McGill University, Montreal, QC Canada

**Keywords:** Canada, Drinking water, Water security, First Nation, Indigenous, Inuit, Métis, Media, Newspaper, Systematic review

## Abstract

**Background:**

Drinking water insecurity and related health outcomes often disproportionately impact Indigenous communities internationally. Understanding media coverage of these water-related issues can provide insight into the ways in which public perceptions are shaped, with potential implications for decision-making and action. This study aimed to examine the extent, range, and nature of newspaper coverage of drinking water security in Canadian Indigenous communities.

**Methods:**

Using ProQuest database, we systematically searched for and screened newspaper articles published from 2000 to 2015 from Canadian newspapers: *Windspeaker*, *Toronto Star*, *The Globe and Mail*, and *National Post*. We conducted descriptive quantitative analysis and thematic qualitative analysis on relevant articles to characterize framing and trends in coverage.

**Results:**

A total of 1382 articles were returned in the search, of which 256 articles were identified as relevant. There was limited coverage of water challenges for Canadian Indigenous communities, especially for Métis (5%) and Inuit (3%) communities. Most stories focused on government responses to water-related issues, and less often covered preventative measures such as source water protection. Overall, Indigenous peoples were quoted the most often. Double-standards of water quality between Indigenous and non-Indigenous communities, along with conflict and cooperation efforts between stakeholders were emphasized in many articles.

**Conclusion:**

Limited media coverage could undermine public and stakeholder interest in addressing water-related issues faced by many Canadian Indigenous communities.

**Electronic supplementary material:**

The online version of this article (doi:10.1186/s12889-017-4164-4) contains supplementary material, which is available to authorized users.

## Background

With less than 0.5% of the world’s population, Canada is fortunate to have 7% of the world’s renewable fresh water [1]. Not all Canadians, however, have equitable access to safe drinking water [2]. For Indigenous peoples in Canada, safe drinking water often remains a challenge, despite federal, provincial, and territorial efforts to address water issues [3–6]. Indigenous peoples is a collective name for the original peoples of North America and their descendants. There are three constitutionally recognized groups of Indigenous peoples in Canada (First Nations, Métis, and Inuit) and all three groups are distinct from each other and have unique histories, languages, cultural practices, and spiritual beliefs. Indigenous communities tend to experience more drinking water security (e.g. access to sufficient and safe water) challenges than non-Indigenous communities in Canada [7, 8].

Water quantity concerns facing some Indigenous communities include challenges in obtaining enough safe water for consumption and basic hygiene. For example, in the Northwest Territories where Indigenous peoples represent 48% of the population, resource development and climate change place pressures on water resources [9]. Water quality has also been challenging for many First Nation [3, 4, 10], Inuit [11–13], and Métis [14, 15] communities. Drinking water advisories are issued when water quality is compromised, and these drinking water advisories are 2.5 times more frequent in First Nation communities compared to non-First Nation communities [5]. Advisories are intended to be a temporary measure to protect public health; however, many First Nation communities experience prolonged periods of drinking water advisories lasting an average of 343 days [16]. As of September 30, 2015, there were 138 drinking water advisories in effect in 94 First Nation communities across Canada [17]. In comparison, it was estimated that there were at least 1838 drinking-water advisories in effect in Canada at the beginning of 2015 [18]. In response to drinking water quality concerns, the Government of Canada commissioned an independent national risk assessment on First Nations water systems in 2011, which found that over 39% of First Nation water systems were “high risk,” indicating that these water systems have major deficiencies and pose a high risk to the quality of water and to human health [19]. Climate change will only further threaten water security for many of these water systems [20]. Despite the Government commissioned assessment and approximately $3 billion invested in water and wastewater since 2006 [21], the challenge persists, with progress reports indicating that, despite improvements, 19% of First Nation water systems are still considered “high risk” [22].

These water quality challenges have important public health implications, resulting in many Canadian Indigenous populations identified as a group at increased risk of waterborne illness compared to Canadian non-Indigenous populations [2, 10, 13, 23, 24]. For instance, untreated water was a suspected source of *Giardia* infection accounting for 29% of notifiable gastrointestinal illness in the Northwest Territories [25]. Furthermore, about a third of residents of the Nunavik Inuit region consume untreated water, placing some Inuit at increased risk for waterborne gastroenteritis [26, 27]. Water quality challenges have also attracted widespread media coverage, including the discovery of *Escherichia coli* in the water supply in Kashechewan, Ontario, where the combination of flooding and contaminated water led to the evacuation of nearly half of the Kashechewan First Nation, illustrating the consequences of insufficient water infrastructure in some Indigenous communities [28, 29].

Water insecurity not only impacts physical health, but can also impact Indigenous peoples spiritual health [30]. For some Indigenous peoples, particularly some First Nation peoples, water has spiritual characteristics and is sometimes considered a spirit; as such, water is often considered sacred and can be important for healing and forming spiritual relationships [31, 32]. Furthermore, water supports Indigenous ways of life for many First Nation [4, 33], Inuit [34–36], and Métis people [15]. As Grand Chief B.G. Cheechoo, Chief of the Nishnawbe-Aski Nation, explains: “Our history is tied to these waters. Our continued reliance on fishing, trapping and hunting and our desire to do so is dependent on these waters. Our future is based on these waters… Any threat to such waters poses a direct threat to our survival” [33].

While research on water security in Indigenous communities is growing, there remains a deficit in studies examining risk perceptions of Indigenous water issues among Indigenous peoples and the general public [37, 38]. Examining risk perceptions can provide insight into understanding individual risk-averting behaviour and management strategy preferences [39–41]. Going further, risk perceptions [42, 43] and public understanding [44] of water-related issues can be shaped by media coverage. For instance, media can shape perceptions through “agenda setting” [45], which is the ability of the media to focus public attention on key topics (e.g. through the number of stories published on a topic) [46]. This focus on key topics, in turn, can influence which issues the public learns about and considers important and worthy of directing public resources towards solving.

Public perceptions are also influenced by the context in which issues are presented in the media [47]. For example, media can frame issues within the context of specific terms, events, or perspectives, through the process of selecting and emphasizing certain elements of a story, as well as the way characters and actors are portrayed [45, 48, 49]. Media coverage of Indigenous issues has been reported to be problematic, as Indigenous issues are often framed with a negative tone rather than a neutral tone – potentially leading the public to have an incomplete, or inadequate picture of the larger issues at play – or, the issues are excluded from media coverage all together [50]. This point has been further emphasized in the 94 Calls to Action put forward by the Truth and Reconciliation Commission of Canada (TRC) [51], with the final report calling on the media for continued, concerted, and “dedicated news coverage and online public information resources on issues of concern to [Indigenous] peoples” (84.3, p. 294). This Call to Action requires that the media take responsibility to ensure reporting of Indigenous peoples and issues in Canada is fair, representative, and non-discriminatory, and that journalists are informed and take responsibility for “learning about the history of [Indigenous] peoples and the issues that affect their lives” (86, p. 295) [51].

Although Indigenous peoples represent 4.3% of the Canadian population [52], water issues disproportionately impact Indigenous communities and these issues can be underrepresented in mainstream media [51]. Indeed, a recent study in 2013 found that news stories on Indigenous issues in Ontario, Canada amounted to 0.46% of all news stories, and had limited coverage of health or water issues [53]. Considering the lack of understanding of how Indigenous water issues are perceived, media’s influence on perceptions, and the TRC’s call for dedicated news coverage for Indigenous issues, the goal of our research is to better understand how media portray water security challenges in Indigenous communities. Specifically, the objectives are to examine the extent, range, and nature of newspaper coverage of drinking water security challenges in Canadian Indigenous communities. By examining newspaper coverage, we can begin to understand the ways in which public opinion and understanding of drinking water security challenges can be shaped, thus providing implications for decision-making and action in addressing water-related challenges, not only in Indigenous communities in Canada but also potentially other Indigenous peoples facing similar challenges globally.

## Methods

### Data sources and search strategy

The search was conducted using ProQuest database for news articles from four major news sources: Canada’s only Indigenous newspaper, *Windspeaker*; Canada’s two national newspapers, *The Globe and Mail* and *National Post*; and Canada’s highest newspaper readership, *Toronto Star* [54]. News articles published from 1st January 2000 to 31st December 2015 were selected. Articles were selected from January 2000 onwards because of the increased public awareness of drinking water security challenges following the outbreak in Walkerton, Ontario (non-Indigenous community) in May 2000 where 7 people died and 2300 became ill, reflected by increased water-related news coverage [44]. Search terms used to search the ProQuest database are presented in Table 1. All citations were imported into EPPI-Reviewer 4 (Eppi-Centre, London, UK), a systematic review software.

**Table 1:** Search strategy to identify news articles on drinking water security in Indigenous communities in Canada

Database	Search terms^a^
ProQuest	("drinking water" OR "tap water")
	AND
	("first nation^*^" OR aboriginal^*^ OR nunavut OR "northwest territor^*^" OR NWT OR Yukon OR nunavik OR nunatsiavut OR amerind^*^ OR inuit^*^ OR inupiat^*^ OR kalaallit^*^ OR aleut^*^ OR metis OR native^*^ OR indian^*^ OR eskimo^*^ OR tribe^*^ OR algonquian OR algonquin OR atikamekw OR blackfoot OR cree OR malecite OR mi’kmaq OR innu OR montagnais OR naskapi OR ojibway OR oji-cree OR michif OR athapaskan OR carrier OR wetsuweten OR chilcotin OR dene OR tlicho OR gwich’in OR slavey OR sarcee OR beaver OR sekani OR kaska OR tahltan OR tuchone OR haida OR iroquoian OR mohawk OR cayuga OR oneida OR kutenai OR salish OR shuswap OR thompson OR halkomelem OR lillooet OR okanagan OR squamish OR straits OR siouan OR dakota OR stoney OR tlingit OR tsimshian OR gitksan OR nisga’a OR wakashan OR nootka OR haisla OR heiltsuk OR kwakiutl OR wakashan OR inuktitut OR inuinnaqtun OR inuvialuktun)

^a^In an attempt to capture the diversity of First Nation groups, language groups from Statistics Canada [103] were used as search terms

### Screening

A two-step relevance screening strategy was employed by two independent reviewers using EPPI-Reviewer 4. In the primary screening, titles and first paragraphs of news articles were screened for relevance. Articles deemed relevant (i.e. explicitly mentioned drinking water and Canadian Indigenous communities) then proceeded to the secondary screening, where the full-text of articles were again screened for relevance (i.e. explicitly mentioned drinking water in Indigenous communities in Canada). Reviewers met throughout the screening process to resolve any conflicts through discussion. To assess the reliability of the relevance screening protocols, an inter-rater reliability agreement, Cohen’s Kappa coefficient, was calculated [55].

### Data analysis

A hybrid deductive and inductive thematic analysis [56, 57] was conducted on the articles identified as relevant in the screening steps. The thematic analysis steps used in this study have been described previously [57]. A mixed-methods software, Dedoose version 6.1.11, was used to assist in data organization and retrieval. Descriptive statistical analyses of frequencies and trends were performed using Stata/IC 13.1.

#### Deductive qualitative analysis

A data extraction form was designed and divided into three sections (See Additional file 1). The first section of the data extraction form was created a priori and captured general, descriptive information identified about each article. The second section of the form captured excerpts of text reflecting deductive concepts identified a priori [56, 57] based on published literature, and included blame, climate change, government response to drinking water security challenges, drinking water infrastructure, regulatory framework, drinking water policy, source water protection or multi-barrier approach to safe drinking water, and Indigenous governance. Finally, each article was coded to capture how the story was framed, including the main focus, the emotional tone or valence [58], episodic or thematic frames used in coverage [59], and substantive or ambiguous content [60] (Table 2).

**Table 2:** Coding type of frame of news articles for deductive qualitative analysis

Framing category	Sub-category	Description	Salient article examples
Focus	Water	Focus on water issues	Title: *Many natives drinking unsafe water.*
			Example: "Walkerton made the news across Canada," the National Chief of the Assembly of First Nations said.
			Source: *The Globe and Mail* May 5, 2001.
	Other	Mentions water issues but focuses on other challenges	Title: *Let the objections finally cease.*
			Example: No child can learn properly if the house she lives in is falling down, the family situation isn't stable and food and water are insufficient or unreliable.
			Source: *The Globe and Mail* Mar 16, 2007.
Valence	Positive	Focus on solutions, or progress towards change	Title: *Natives shouldn't have to boil their water.*
			Example: This government will ensure that community leaders have what they need to deliver clean water to their residents.
			Source: *National Post* Mar 23, 2006.
	Negative	Focus on problems, lack of action, or blame	Title: *Natives deserve better.*
			Example: I’m absolutely disgusted to hear that tainted water continues to plague Canada’s First Nations Reserves.
			Source: *National Post* Oct 29, 2005.
	Neutral	Neither a positive nor a negative valence	Title: *Lessons from Kashechewan.*
			Example: In October, the story broke that the village's 1,900 Cree were drinking water polluted with *E. coli.*
			Source: *National Post* Nov 21, 2005.
Episodic and thematic	Episodic	Focus on a specific event or topic, such as a waterborne outbreak or a new legislation	Title: *Ontario evacuating reserve.*
			Example: Ontario's minister for Aboriginal Affairs, David Ramsay, said the province will evacuate more than half of the residents of Kashechewan First Nation after the reserve's water supply tested positive for *E. coli.*
			Source: *National Post* Oct 26, 2005.
	Thematic	Focus on a theme, such as drinking water issues in general	Title: *Water on reserves a problem, group says.*
			Example: The drinking-water problems on Canada’s native reserves need to be addressed immediately, a roundtable on the subject concluded yesterday.
			Source: *The Globe and Mail* July 18, 2001.
Content	Substantive	Use specific information	Title: *Clean water plan unveiled for reserves.*
			Example: 170 of 755 water treatment systems pose health hazards due to lack of training, maintenance and standards.
			Source: *Toronto Star* Mar 22, 2006.
	Ambiguous	Use vague information	Title: *Reserve conditions worsening, overhaul needed, feds told.*
			Example: The report found education, housing, child welfare and access to safe drinking water remain major problems on First Nations reserves.
			Source: *Toronto Star* Jun 10, 2011.

#### Inductive qualitative analysis

Through an inductive or ‘bottom up’ approach, the codes derived were data-driven and emerged from the information articled. We focused on analyzing information presented through quotations, including information about environmental issues, financial issues, health outcomes, and infrastructure or training issues (Table 3). Data collected included the types of sources quoted, what sources said about the issue, and in what context. While the content of direct quotes were analysed, and the length of quotes was not considered in the data extraction.

**Table 3:** Data-driven codes derived from sources quoted for inductive qualitative analysis

Major issues discussed	Example of source(s) quoted	Salient article examples
Environmental	Non-Indigenous Politician	Title: *Life on some reserves ‘unacceptable’, minister says.*
		Example: The living conditions on some Ontario first nation reserves, where contaminated land has forced several communities to close their local schools, are "offensive" and "unacceptable," Ontario Aboriginal Affairs Minister Michael Bryant said yesterday.
		Source: *The Globe and Mail* Nov 16, 2007.
Financial	Elected Indigenous person (elected)	Title: *Ottawa to give reserves $600-million.*
		Example: Mr. [Ken Young] said the two most pressing concerns he would like to see addressed tomorrow is money for housing and health care for First Nations - Ken Young Vice-Chief of Assembly of First Nations.
		Source: *National Post* Feb 17, 2003.
Health outcomes	Indigenous person (non-elected)	Title: *Indian reserves face threat from contaminated water.*
		Example: "Our children and our elders have suffered frequent illness including a recent outbreak of gastroenteritis," Chief Wilfred Wesley of Cat Lake Reserve.
		Source: *Toronto Star* April 16, 2003.
Infrastructure or training	Academic	Title: *Dirty water.*
		Example: "The boil water advisory is being used as an alternative to [water] treatment" Steve Hrudey of University of Alberta.
		Source: *National Post* Apr 9, 2008.

## Results

**Fig. 1:**
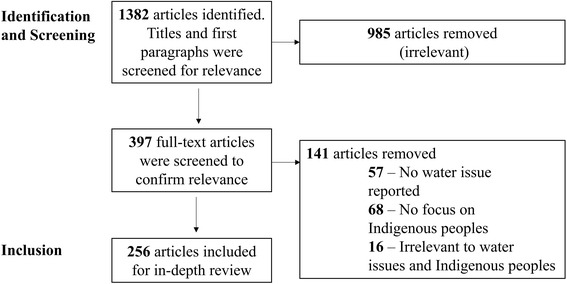
Selection of newspaper articles that examined drinking water security in Canadian Indigenous communities

The initial search retrieved 1382 articles. After primary screening, 397 article’s full-text were reviewed, and 256 articles were identified as relevant and retained for analysis (Fig. 1). The primary screening and secondary screening by two independent reviewers achieved a kappa value of 0.64 and 0.67, respectively, indicating “substantial agreement” in coding between the two reviewers according to Landis and Koch’s interpretations of kappa values [55]. The *Toronto Star* published the most articles (34%), followed by *The Globe and Mail* (31%), *National Post* (20%) and *Windspeaker* (15%) (Table 4). Almost 15% of articles were published on the front page. The majority of stories published were news articles (75%), followed by editorials (11%), opinion/column (9%), and letters to editor (5%). Articles were mainly authored by journalists (87%), and scientific evidence (i.e. reference to a report or study) was provided in 83 articles (32%). Coverage focused primarily on issues in First Nations communities (92% of all articles), and some articles used the umbrella term “Aboriginal” to describe challenges among all Indigenous groups (10%). Very few articles covered issues specific to Inuit (3%) and Métis (5%) communities.

**Table 4:** Descriptive information extracted from Canadian news articles on Indigenous drinking water security from 2000-2015

Description	n (% of articles in sample)
Newspaper	
* Toronto Star*	88 (34)
* The Globe and Mail*	79 (31)
* National Post*	52 (20)
* Windspeaker*	38 (15)
Word count	
1-250	37 (14)
251-500	73 (28)
501-1000	125 (49)
1001+	22 (9)
Article type	
News	192 (75)
Editorial	29 (11)
Opinion or column	23 (9)
Letter to editor	13 (5)
Author	
Journalist	223 (87)
Other	34 (13)

### Most articles focused on drinking water governance challenges

**Fig. 2:**
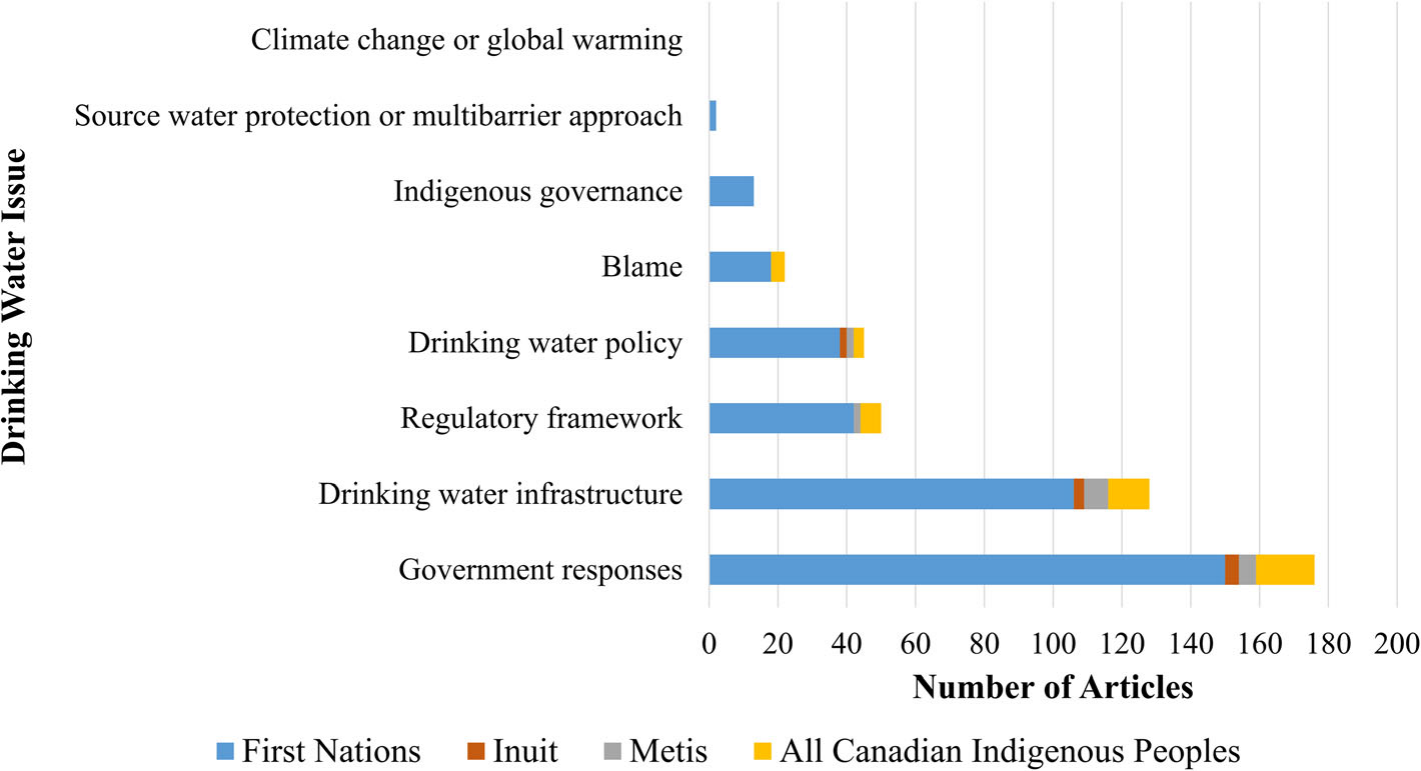
Number of articles covered by Canadian newspapers on Indigenous drinking water security from 2000-2015

**Fig. 3:**
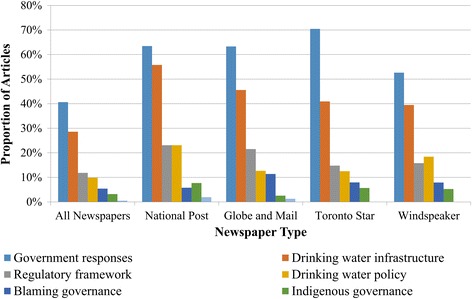
Drinking water security among Canadian Indigenous communities covered by Canadian newspapers from 2000-2015

The majority of news articles focused on drinking water security challenges (71%), and almost exclusively focused on drinking water quality. Other articles that briefly mentioned drinking water focused on other social or economic challenges, as well as inequities faced by Indigenous communities (29%). Points of discussion surrounding drinking water security most often included government responses (64%), followed by drinking water infrastructure (45%), regulatory frameworks (19%) and drinking water policy (16%) (Fig. 2), with coverage of water-related challenges relatively consistent across newspapers (Fig. 3). There was low story reference to blame (9%), Indigenous governance (5%), source water protection/multi-barrier approach to safe drinking water (1%), and climate change (0%). Note that there could be more than one issue discussed in each article; as such, these categories were not mutually exclusive.

### Indigenous voices focused on drinking water challenges

**Fig. 4:**
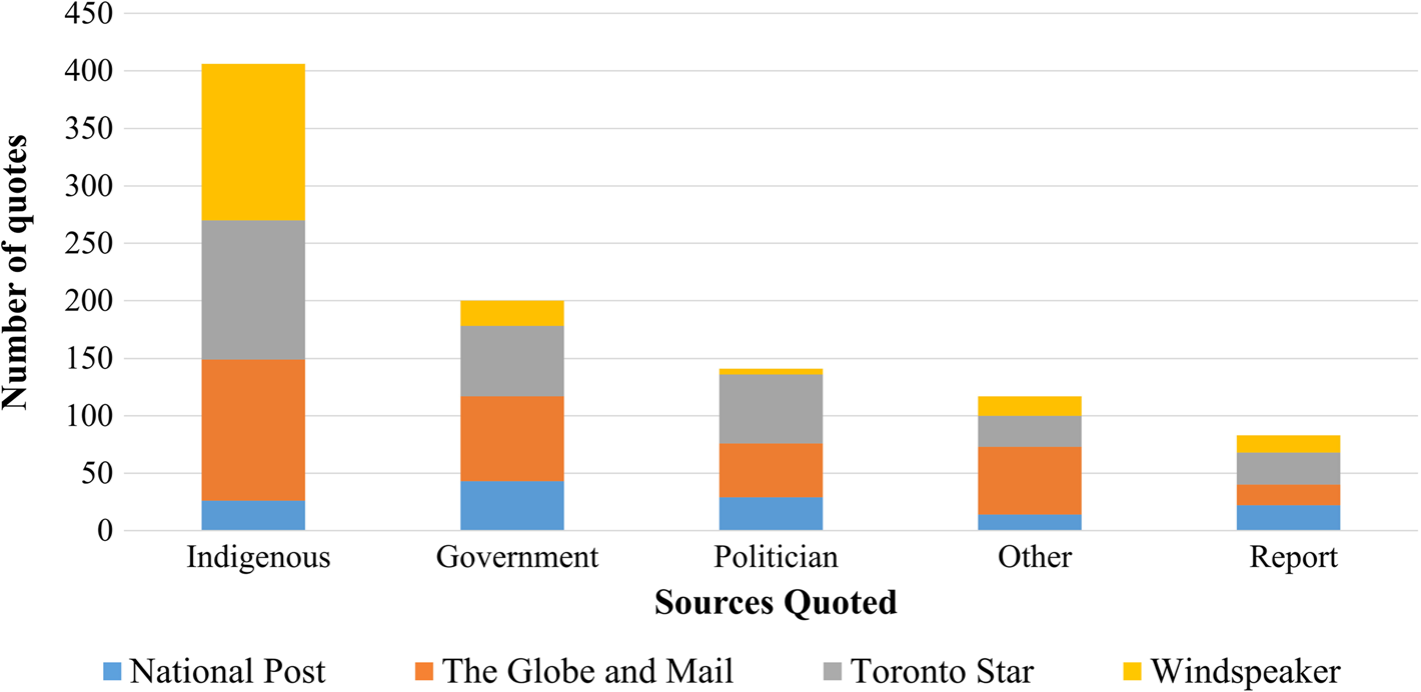
Sources quoted on Canadian Indigenous drinking water security covered by Canadian newspapers from 2000-2015

All newspapers quoted Indigenous peoples more often than non-Indigenous peoples, except for the *National Post* where government sources were the most quoted group (Fig. 4). Out of all Indigenous peoples quoted between 2000 and 2015, Indigenous elected officials (e.g. Chiefs and Band Councilors) were quoted most often, representing 76% of all quotations from Indigenous peoples. Scientists or academics were consistently the least-quoted source across newspapers and time period.

Government representatives often justified government spending. For example, in 2008, the former Indigenous and Northern Affairs Canada (INAC) Minister Chuck Strahl indicated “we want First Nations to have increased confidence in the quality of their drinking water” in regard to the government’s new $330 million funding for a drinking-water plan [61]. Conversely, Indigenous peoples, both in elected positions and non-elected positions, often expressed concerns regarding: inadequate training; health implications of water challenges; natural environment problems such as contamination of surface water [62]; regulatory concerns; and inadequate infrastructure. For instance, in 2007, the former National Chief of the Assembly of First Nations, Phil Fontaine, stated “the problems plaguing hundreds of First Nations who continue to be under boil-water advisories are well known. Yet, for First Nations, the budget contains absolutely no money but promises a tough regulatory regime and standards for drinking water on First Nation reserves" [63]. Other Indigenous peoples raised similar concerns, including Ashley Iserhoff of the James Bay Cree in 2000: “Our people still mostly live in desolate communities with unsafe drinking water and inadequate sanitation. Our people are still crowded into unsafe and unhealthy dwellings or live homeless on the streets of the big cities” [64].

**Fig. 5:**
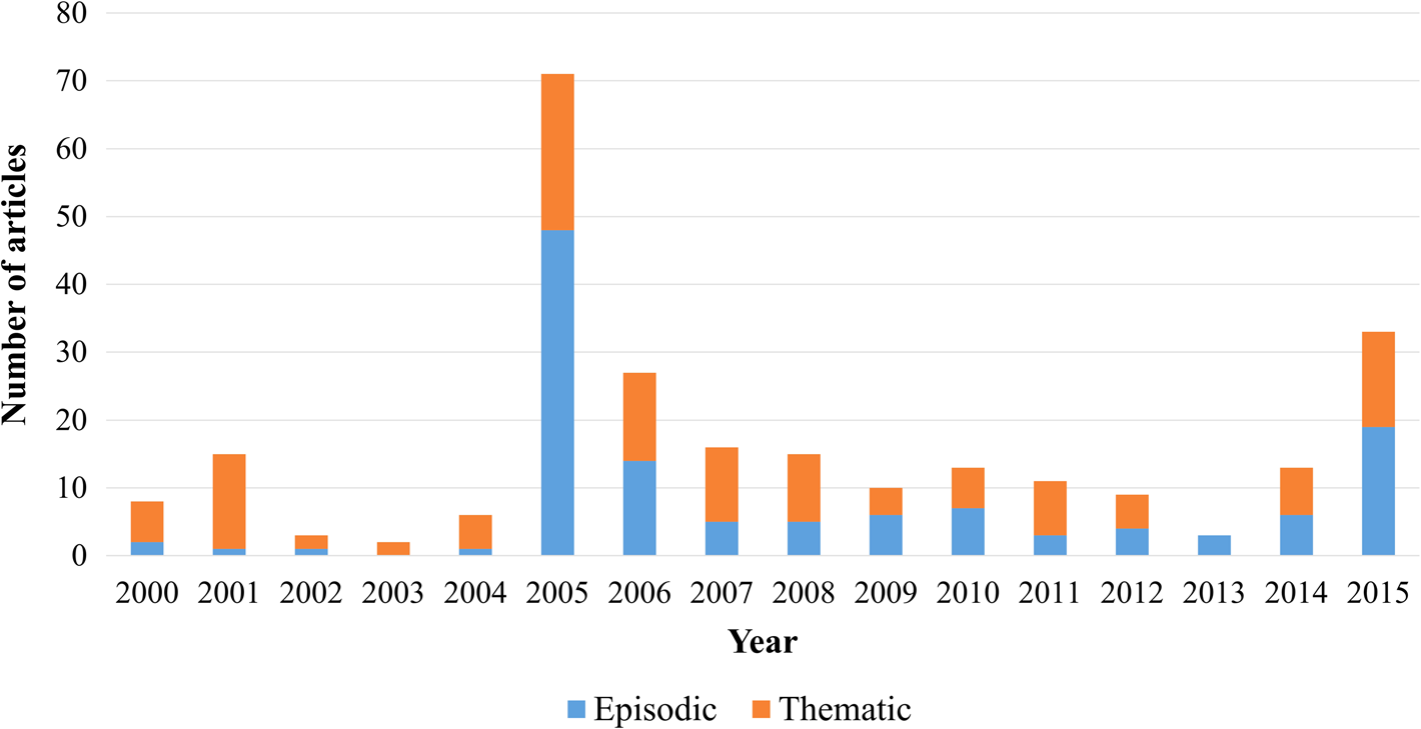
Episodic and thematic framing in Canadian newspaper articles on Indigenous drinking water security from 2000-2015

**Fig. 6:**
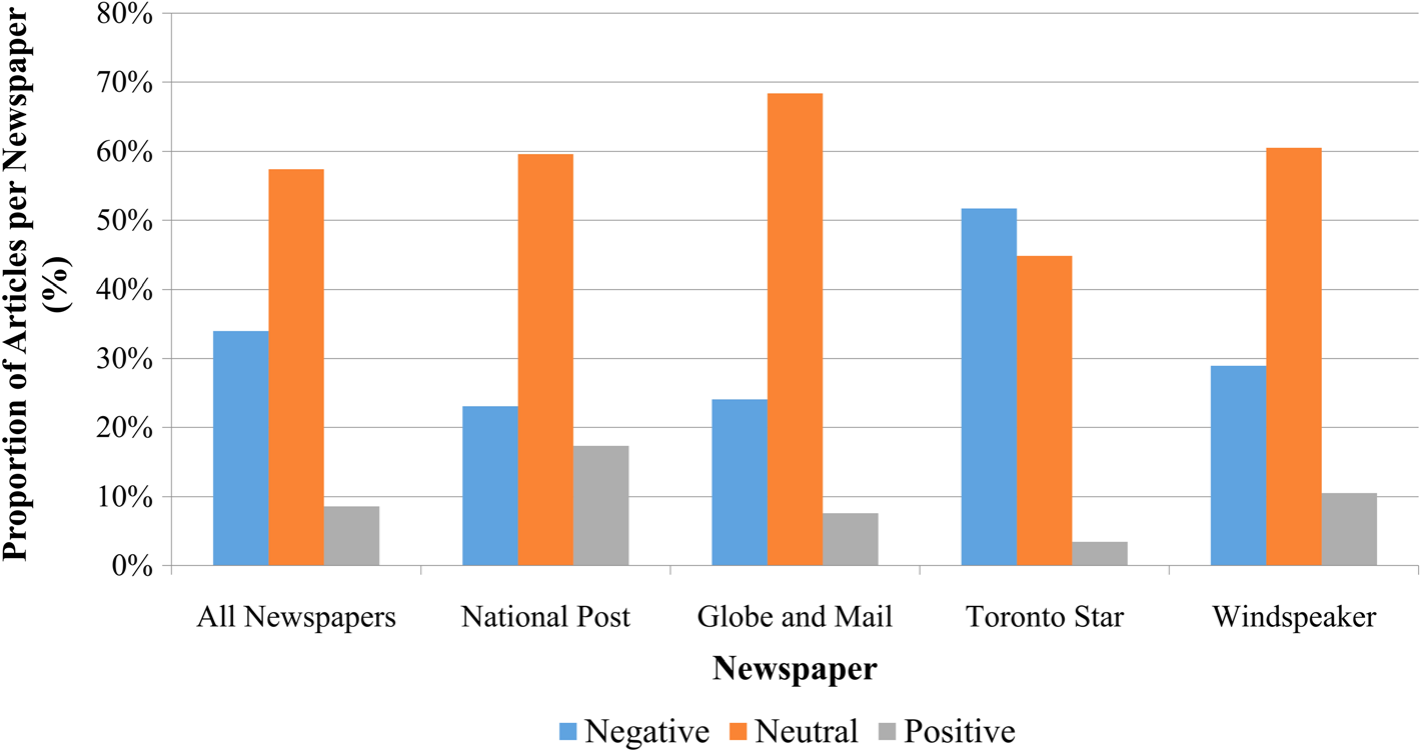
Valence of Canadian news articles on drinking water security among Indigenous communities from 2000-2015

**Fig. 7:**
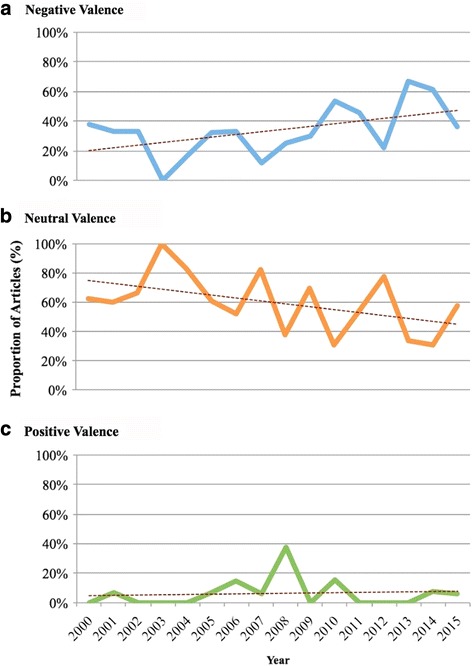
Negative (**a**), neutral (**b**), and positive (**c**) valence of articles on Indigenous drinking water security from 2010-2015

Episodic (48%) and thematic framing (52%) were presented in news articles (Fig. 5). Out of the episodic frames, the majority (65%) focused on the Kashechewan water crisis. Other episodic events (35%), such as a water treatment failure in Constance Lake First Nation [65], received much less media attention. Thematic frames reflected more general, ongoing issues facing Canadian Indigenous communities (52%). Most newspapers framed articles with a neutral valence (58%), followed by negative (32%) and positive (10%) valences, with the exception of *Toronto Star* where news articles were mostly framed with a negative valence (Fig. 6). From 2000 to 2015, negative valence articles increased over time (Fig. 7). Indigenous peoples were the most frequently quoted group in articles with a neutral (39%) or negative valence (40%), whereas government representatives were the most frequently group in articles with a positive valence (40%).

### Focus on conflict and cooperation between stakeholders

Sources often discussed conflicts among groups when discussing various drinking water security challenges. In general, when conflicts with stakeholder groups arose, it was usually in the context of actions and challenges in improving drinking water quality. For example, former Ontario New Democrat Leader Howard Hampton spoke about the Liberal government’s lack of spending: “there is an epidemic of bad water on First Nations…will you stop the platitudes and stop telling First Nations you feel their pain, and start making some financial investments to improve the quality of water in their communities?” [66]. Some conflicts were discussed multiple times over the years; for example, the challenge of improving drinking water infrastructure was mentioned in 2001, 2006, and 2010 [67–69]. For issues surrounding drinking water policy and governmental responsibilities, fragmented jurisdiction for ensuring safe drinking water provision was a concern; for instance, former Liberal Member of Parliament Clifford Lincoln explained in 2001: “the patchwork of drinking water standards across the country is a recipe for disaster…all three levels of government must seek consensus on enforceable standards” [70].

Some news articles commented on media coverage of Indigenous issues compared to the coverage of non-Indigenous issues: “this double standard – extreme public and media concern over Walkerton and little to no concern over lack of safe drinking water on a huge number of aboriginal communities – is deeply disturbing” [71]. Some articles covered cooperation efforts between First Nation groups and levels of government. Speaking to an example of this cooperation, Ian Corbin, former INAC spokesperson, explained “similar to what it is off-reserve, water is a shared responsibility… INAC, Health Canada, First Nations and the private sector all have a role” [72]. Corbin added that the department, in conjunction with Health Canada, Environment Canada, and First Nations, is working on an integrated and insectoral “First Nation water management strategy”.

### Scarce coverage up until the Kashechewan water crisis

Two periods of interest can be discerned from the newspaper coverage: 1) limited coverage from 2000 to 2005; and 2) an increase and subsequently steady coverage from 2005 to 2015 (Fig. 5). The articles published in 2001 and 2005 represented 7% and 29% of articles, respectively. In 2001, the coverage was primarily in response to a waterborne outbreak in Walkerton, Ontario in May 2000, and a waterborne outbreak in North Battleford, Saskatchewan in April 2001. Then, the majority of articles published in 2005 used episodic framing to describe the Kashechewan, Ontario water crisis. Higher-than-average levels of newspaper reporting of drinking water security challenges were sustained afterwards. The increase in coverage in 2015 covered ongoing drinking water advisories on some First Nation reserves, as well as dialogue during the Canadian Federal election in October 2015. For example, one article stated “the next federal government should do an immediate audit of every troubled reserve system. It should then work directly with communities to fix the worst cases, and move on to the less urgent ones after that” [73]. Another article commented that Liberal Leader Trudeau has “pledged to implement all 94 recommendations of the Truth and Reconciliation Commission… [Trudeau] also said he would address the problem of water supply on reserves, but not with a specific itemized promise or timeline” [74].

## Discussion

News coverage of water security challenges in Indigenous communities in Canada from 2000-2015 were examined in this study. Over the 16-year study period, 256 news stories appeared in four prominent newspapers (*Toronto Star, The Globe and Mail, National Post*, and *Windspeaker*). When our results are compared to other studies, our results suggest limited coverage compared to non-Indigenous water challenges; for example, while we found 131 articles in *The Globe and Mail* and the *National Post* over 16 years spanning First Nations, Inuit, and Métis communities across the country, news coverage of the Walkerton water crisis was covered in 652 news stories in the same newspapers over two years [44]. Indeed, a recent study also found that Indigenous populations in Canada are widely underrepresented in mainstream media [53] and, within this coverage, Inuit and Métis water challenges received even less coverage. This finding may be explained by the lack of publically available and accessible data on boil water advisories experienced by Inuit and Métis communities compared to First Nations communities, where boil water advisories are compiled and provided on a monthly basis on Health Canada’s website [75]. Going further, there is a lack of peer-reviewed literature on Inuit or Métis water-related health issues [13, 76–78], reflecting the lack of data collected on Inuit or Métis health issues in general [8]. It is recommended that water and health issues in Inuit and Métis communities are studied further, and regularly compiled and reported to the public.

Government representatives and Indigenous peoples were the primary sources quoted, with both often portraying drinking water quality as ‘shameful’, given that some First Nation communities have been under boil-water advisories for years. Literature have also reflected conditions on First Nation reserves as ‘unacceptable’ [79, 80]. From 2000 to 2015, the number of Indigenous peoples quoted increased, perhaps due to the cumulative impacts of persistent drinking water issues and boil-water advisories [17]. However, the number of quotes alone may not be an adequate indicator of representation of voice in the media. In future studies, considering the length of quotes could provide information on the extent of contextual background information provided, and thus insights into voice representation [81].

Coverage appeared low except for in 2001 and 2005 when news coverage slightly increased due to large waterborne disease outbreak in North Battleford, Saskatchewan (April 2001) and substantially increased in response to water crisis in Kashechewan, Ontario (October-November 2005) (Fig. 5). From this trend, it appeared that media coverage was prominent during and immediately after a water contamination event, but was not well covered before the event despite the chronic and on-going water security challenges in many communities. Further, despite the importance of preventative measures for safe drinking water, such as multi-barrier protection [82], or source water protection [5], these preventative measures were rarely reported in news articles. As such, news coverage focused more on responses and recovery rather than prevention and mitigation. This finding is supported by a recent media analysis of Canadian Indigenous issues, that found government responses generally followed after concerns were voiced or crises were experienced, rather than before such incidents [53]. The low coverage on prevention and high focus on responses could be explained by media’s interest in government actions and agendas [83, 84], and is consistent with other studies where coverage on government responses was high, for example during the Walkerton, Ontario water crisis [44]. Considering preventative measures have potential to improve drinking water quality for Indigenous communities in Canada [5], future media messaging with the help of public health practitioners and water stakeholders, could focus more on prevention and mitigation to protect water security in Indigenous communities.

The Kashechewan, Ontario water crisis in 2005, a short-term event, gained substantial coverage compared to communities experiencing long-term boil water advisories, consistent with studies where novel risks experienced over a short time frame generally had higher coverage than longer term risks [85]. However, it is important to note that water issues in Kashechewan are ongoing; for example, Kashechewan recently experienced floods leading to the evacuation of some of its residents in 2014 and 2015 [86]. With limited media coverage on Indigenous water security, except during a large outbreak, smaller or chronic waterborne outbreaks may be overlooked by politicians, decision makers, and the general public. To overcome this challenge, public health providers and practitioners may play an important leadership role in developing media relationships to promote coverage of communities with long-term drinking water issues [83, 87].

Most articles focused on government responses to Indigenous water security challenges, and in particular, focused on inadequate government spending and action. The lack of government funding available to address Indigenous water security challenges [82, 88, 89], along with the history of water-related government actions [82, 89], have been reported in peer-reviewed literature. While progress was made in improving safe drinking water provision in high risk communities [90], the emphasis on government responses surrounding inadequate funding and action, along with the persistence of drinking water issues [75], suggest a call for further government support and action.

Governance challenges reported in the articles reviewed included: drinking water policy challenges; infrastructure challenges including technical and financial; and regulatory framework challenges. Among articles that focused on drinking water policy challenges, articles discussed the lack of legally enforceable Federal safe drinking water standards on reserves [91], along with the complicated policy framework currently in place [88]. Articles that focused on infrastructure challenges primarily discussed challenges with water treatment infrastructure, the lack of local operator training, and the lack of financial support, consistent with challenges reported in literature [88, 92]. News coverage on regulatory framework challenges mainly focused on the complicated jurisdictional issues regarding water on First Nations reserves [6, 89, 93], highlighting the difficulties when the responsibility for water quality on reserves is shared between First Nations councils and the Federal government [89] and provincial governments are responsible for regulating and enforcing legislation and policies relating to municipal water systems.

Disproportionate government efforts in addressing water security challenges between Indigenous and non-Indigenous communities was emphasized in news articles. For example, a large waterborne disease outbreak occurred in Canada (Walkerton, Ontario in May-June 2000). This event brought considerable media coverage and attention to water quality issues in Canada, and led to policy changes, new regulations (e.g. *Safe Drinking Water Act, 2002*), and public inquiries to address such issues at Provincial, Territorial, and Federal scales within two years after the outbreak [94, 95]. These new policies, however, do not apply to, and do not jurisdictionally impact, First Nation reserves [96]. The *Safe Drinking Water for First Nations Act* was introduced in 2013 [97], eight years after the 2005 Kashechewan water crisis and other ongoing water issues. This delayed action suggests the “double standard” that Indigenous peoples emphasized in news articles, which may be responsible for the differences in water security experienced among different groups across the Nation [82].

Indigenous water security was largely covered using a neutral or a negative emotional tone, consistent with negative tone framing for other environmental issues such as natural disasters [83] and climate change [98]. Articles with negative tones focused on government responses to Indigenous water security challenges, specifically on inadequate government spending or action. Articles with a positive tone focused on solutions such as introducing new government budgets, perhaps for the purpose of advancing and/or supporting political agendas [84]. It is likely that the media representation of drinking water will affect the way that actions are taken. The high negative coverage of articles on inadequate government responses is likely to promote this negative image to the public, and the public may be aware that drinking water is problematic and requires government responses to mitigate this problem. Articles also represented a nearly equal balance of episodic and thematic frames. Past research suggested that episodic frames tend to present events as isolated incidents, and in the absence of the broader context, can lead to shallower understanding of political and social issues [59, 99]. Coverage using thematic framing generally reflected the chronic Indigenous water security issues. Research suggests that thematic frames generally help readers view a topic as an ongoing issue, rather than a one-time event, and are more likely to cause people to see an issue as a shared responsibility [59]. As both frames were represented, readers would likely expect some action taken by decision-makers to address Indigenous water security challenges.

Additionally, the themes of conflict and cooperation emerged prominently throughout the analysis. Jurisdictional conflicts surrounding drinking water exist [6, 96], and cooperation and collaboration among Indigenous groups, Federal government agencies, and provincial governments have been acknowledged as essential for moving forward with drinking water safety [82, 96]. Many factors contributed to the rise in tension, in particular, the Kashechewan water crisis, as well as the chronic boil water advisories in many First Nation communities. Articles often leaned towards conflict likely to draw readers, consistent with other studies where conflict frames were prominent in media coverage of environmental issues such as climate change [43, 100, 101]. Interestingly, climate change framing was absent in news articles, despite the impact of climate change on water security [12, 13, 102]. It is recommended that news articles begin to discuss water issues in the context of climate change.

This study provided a comprehensive and systematic review, however, there are several important study limitations. While “substantial agreement” in relevance screening was achieved, the reviewers faced some difficulties as many articles had only vague reference to water issues. This study also did not examine other media that might also be influential in shaping public perceptions such as radio or television; however, we reported on a selection of Canadian newspapers with the highest circulation figures, along with the *Windspeaker* (leading Indigenous news source in Canada), which likely provided a good reflection of overall news coverage in Canada from 2000-2015. Coverage in non-Indigenous communities was also not examined; as such, direct comparisons between water security coverage in Indigenous and non-Indigenous communities were not possible, but may have provided more insight into the equity of coverage between groups. Although our results suggest that Indigenous water security issues are under-represented in the media, further research with non-Indigenous comparison groups should be conducted to test this hypothesis. We also analyzed framing in coverage and key themes but we do not know how this information is received and interpreted by different audiences. Further research is needed to determine how coverage compares between Indigenous and non-Indigenous communities in Canada; how coverage differs from other sources of media; and how decision-makers and the general public perceive water security challenges covered by the media.

## Conclusion

Any combination of media attention, public inquiry, and new legislation can be expected when water security issues emerge, as in the case of the Walkerton, Ontario water crisis; however, the same level and timeliness of response was absent for Indigenous communities, as in the case of Kashechewan, Ontario water crisis. This research indicates that coverage of Indigenous water security over the 16-year time period appeared to be limited and inequitable. Furthermore, there was also very limited coverage of water security in Inuit or Métis communities, with coverage focusing mainly on responses to water quality issues rather than prevention and mitigation. In a post-Truth & Reconciliation Commission era, this inequitable and inadequate coverage is simply not acceptable, and we support the TRC’s (2015) recommendations for action that call on the media to have continued, dedicated, and equitable news coverage on issues of concern to Indigenous peoples – and indeed, all Canadians – moving forward (84.3), and to develop resources and strategies that can inform and educate the public (85.2). Closing the media reporting gap can be a positive step towards closing the water security gap and related health outcomes between Canadian communities.

## Additional file

Additional file 1: Descriptive information, drinking water issues, and framing of articles extracted from articles for deductive qualitative analysis. (DOCX 15 kb)

## Abbreviations

INAC: Indigenous and Northern Affairs Canada
TRC: Truth and Reconciliation Commission of Canada

## References

[1] Environment Canada: Drinking water questions. 2012. http://www.ec.gc.ca/eau-water/default.asp?lang=En&n=1C100657-1. Accessed 26 Sep 2014.

[2] Adelson N (2005). The embodiment of inequity: health disparities in Aboriginal Canada. Can J Public Health.

[3] Eggertson L (2008). Despite federal promises, first nations’ water problems persist. Can Med Assoc J.

[4] Eggertson L (2006). Safe drinking water standards for First Nations communities. Can Med Assoc J.

[5] Patrick RJ (2011). Uneven access to safe drinking water for First Nations in Canada: Connecting health and place through source water protection. Health Place.

[6] Swain H, Louttit S, Hrudey S: Report of the expert panel on safe drinking water for First Nations, Volume 1. 2006. http://publications.gc.ca/site/eng/298371/publication.html. Accessed 3 Jan 2016.

[7] Metcalfe C, Murray C, Collins L, Furgal C (2011). Water quality and human health in indigenous communities in Canada. Glob Bioeth.

[8] Health Council of Canada: The health status of Canada’s First Nations, Metis and Inuit peoples. 2005. http://publications.gc.ca/collections/collection_2012/ccs-hcc/H174-37-2005-1-eng.pdf. Accessed 18 Feb 2015.

[9] Indigenous and Northern Affairs Canada: Water Today: Water Quality and Quantity in the NWT. 2010. http://www.aadnc-aandc.gc.ca/DAM/DAM-INTER-NWT/STAGING/texte-text/ntr_pubs_wt10_1330704074233_eng.pdf. Accessed 19 Feb 2016.

[10] Harbinson M: An Analysis of Water Quality and Human Health Issues in First Nations Communities in Canada. 2012 http://qspace.library.queensu.ca/bitstream/1974/7074/1/ENSC501_FinalReport1_M.Harbinson.pdf. Accessed 8 Jan 2016.

[11] Sarkar A, Hanrahan M, Hudson A: Water quality in Aboriginal communities in Labrador: a study of the Southern Inuit community of Black Tickle. St John’s, NL. 2015. https://www.mun.ca/harriscentre/reports/Sarkar_Water_12_13_Final.pdf. Accessed 4 April 2016.

[12] Martin D, Bélanger D, Gosselin P, Brazeau J, Furgal C, Déry S (2007). Drinking water and potential threats to human health in nunavik: Adaptation strategies under climate change conditions. Arctic.

[13] Harper SL, Edge VL, Schuster-Wallace CJ, Berke O, McEwen SA (2011). Weather, water quality and infectious gastrointestinal illness in two Inuit communities in Nunatsiavut, Canada: Potential implications for climate change. Ecohealth.

[14] Bentley D, Murphy B (2006). Power, praxis and the métis of Kelly lake, Canada. Can J Native Stud.

[15] Szach NJ: Keepers of the water: Exploring Anishinaabe and Metis women’s knowledge of water and participation in water governance in Kenora, Ontario. 2013. https://www.umanitoba.ca/institutes/natural_resources/Left-Hand Column/theses/Masters Thesis Penneys-Szach 2013.pdf. Accessed 8 Jan 2016.

[16] Health Canada. Drinking water advisories in First Nations communities in Canada. A National Overview 1995–2007. 2009. www.hc-sc.gc.ca/fniah-spnia/pubs/promotion/_environ/2009_water-qualit-eau-canada/index-eng.php. Accessed 3 Jan 2016.

[17] Health Canada: Drinking Water Advisories in First Nations Communities. 2015. http://www.hc-sc.gc.ca/fniah-spnia/promotion/public-publique/water-dwa-eau-aqep-eng.php. Accessed 3 Jan 2016. 10.3390/ijerph13050505 PMC4881130 27196919

[18] Lui E. On Notice for a Drinking Water Crisis in Canada. 2015. http://canadians.org/sites/default/files/publications/report-drinking-water-0315.pdf. Accessed 3 Jan 2016.

[19] Neegan Burnside Ltd: National Assessment of First Nations Water and Wastewater Systems: National Roll-Up Report Final. 2011. http://www.aadnc-aandc.gc.ca/DAM/DAM-INTER-HQ/STAGING/texte-text/enr_wtr_nawws_rurnat_rurnat_1313761126676_eng.pdf. Accessed 8 Jan 2016.

[20] Ford JD (2012). Indigenous health and climate change. Am. J. Public Health.

[21] Hyslop K: A fresh fix for unsafe water on First Nations reserves. 2014. http://thetyee.ca/News/2014/01/13/Water-on-Reserves/. Accessed 24 Mar 2016.

[22] Indigenous and Northern Affairs Canada: Water and Wastewater Infrastructure Investment Report : April 2012 - March 2013. 2015. https://www.aadnc-aandc.gc.ca/eng/1403198954861/1403199074561. Accessed 8 Jan 2016.

[23] Ford JD, Berrang-Ford L, King M, Furgal C (2010). Vulnerability of Aboriginal health systems in Canada to climate change. Glob Environ Chang.

[24] Harper SL, Edge VL, Ford J, Thomas MK, Pearl DL, Shirley J (2015). Acute gastrointestinal illness in two Inuit communities: burden of illness in Rigolet and Iqaluit, Canada. Epidemiol Infect.

[25] Pardhan-Ali A, Wilson J, Edge VL, Furgal C, Reid-Smith R, Santos M (2012). A descriptive analysis of notifiable gastrointestinal illness in the Northwest Territories, Canada, 1991-2008. BMJ Open.

[26] Messier V, Lévesque B, J.F. P, Ward BJ, Libman M, Couillard M, et al: Zoonotic diseases, drinking water and gastroenteritis in Nunavik: a brief portrait. 2007. https://www.inspq.qc.ca/pdf/publications/656_esi_maladies_infectieuses.pdf. Accessed 8 Jan 2016.

[27] Messier V, Lévesque B, Proulx JF, Rochette L, Serhir B, Couillard M (2012). Seroprevalence of Seven Zoonotic Infections in Nunavik, Quebec (Canada). Zoonoses Public Health.

[28] CBC: Water purification unit heads to Kashechewan. 2005. http://www.cbc.ca/news/canada/water-purification-unit-heads-to-kashechewan-1.549135. Accessed 26 Nov 2014.

[29] CBC: Kashechewan: water crisis in Northern Ontario. 2006. http://www.cbc.ca/news2/background/aboriginals/kashechewan.html. Accessed 26 Nov 2014.

[30] Environment and Climate Change Canada: Water and Canada’s Aboriginal Peoples. 2010. https://www.ec.gc.ca/eau-water/default.asp?lang=En&n=BA5125BF-1. Accessed 19 Feb 2016.

[31] Anderson K: Aboriginal Women, Water and Health: Reflections from Eleven First Nations, Inuit, and Métis Grandmothers. 2010. http://www.onwa.ca/upload/documents/womenandwater.pdf. Accessed 24 Mar 2016.

[32] Kim A, Barbara C, Margaret HB (2013). Carriers of water: Aboriginal women’s experiences, relationships, and reflections. J Clean Prod.

[33] Schelwald-van der Kley L, Reijerkerk L, Bakker K (2009). Water. A way of life: Sustainable water management in a cultural context.

[34] Goldhar C, Bell T, Wolf J (2014). Vulnerability to freshwater changes in the Inuit settlement region of Nunatsiavut, Labrador: A case study from Rigolet. Arctic.

[35] Cunsolo Willox A, Harper SL, Edge VL, Landman K, Houle K, Ford JD (2013). The land enriches the soul: On climatic and environmental change, affect, and emotional health and well-being in Rigolet, Nunatsiavut, Canada. Emot Sp Soc.

[36] Cunsolo Willox A, Harper SL, Ford JD, Landman K, Houle K, Edge VL (2012). “From this place and of this place:” Climate change, sense of place, and health in Nunatsiavut, Canada. Soc Sci Med.

[37] Dupont D, Waldner C, Bharadwaj L, Plummber R, Carter B, Cave K (2014). Drinking water management: health risk perceptions and choices in First Nations and non-First Nations communities in Canada. Int J Environ Res Public Health.

[38] Ekos Research Associates. Perceptions of drinking water quality in First Nations communities and general population. 2011. http://www.ekospolitics.com/articles/015-11.pdf. Accessed 26 Nov 2014.

[39] Peacock WG, Brody SD, Highfield W (2005). Hurricane risk perceptions among Florida’s single family homeowners. Landsc Urban Plan.

[40] Steg L, Sievers I (2000). Cultural theory and individual perceptions of environmental risks. Environ Behav.

[41] Slovic P (1987). The perception of risk. Science.

[42] Lomborg B (2001). The skeptical environmentalist: Measuring the real state of the world.

[43] Boykoff MT (2009). We speak for the trees: media reporting on the environment. Annu Rev Environ Resour.

[44] Driedger SM (2007). Risk and the media: a comparison of print and televised news stories of a Canadian drinking water risk event. Risk Anal.

[45] McCombs ME, Shaw DL (1972). The agenda-setting function of mass media. Public Opin Q.

[46] Mazur A (1998). Global environmental change in the news: 1987-90 vs 1992-6. Int Sociol.

[47] Scheufele DA, Shanahan J, Kim S-H (2002). Who cares about local politics? media influences on local political involvement, issue awareness, and attitude strength. J Mass Commun Q.

[48] Bennet W (2002). News: the politics of illusion.

[49] Semetko HA, Valkenburg PM (2000). Framing european politics: a content analysis of press and television news. J Commun.

[50] Harding R (2005). The media aboriginal people and common sense. Can J Native Stud.

[51] The Truth and Reconciliation Comission of Canada: Honouring the Truth, Reconciling for the Future: Summary of the Final Report of the Truth and Reconciliation Commission of Canada. 2015. http://www.trc.ca/websites/trcinstitution/File/2015/Findings/Exec_Summary_2015_05_31_web_o.pdf. Accessed 19 Feb 2016.

[52] Statistics Canada. Aboriginal peoples in Canada: First Nations people, Metis and Inuit. 2011. http://www12.statcan.gc.ca/nhs-enm/2011/as-sa/99-011-x/99-011-x2011001-eng.cfm. Accessed 19 Feb 2016.

[53] Journalists for Human Rights. Buried Voices : Media Coverage of Aboriginal Issues in Ontario. 2013. http://www.jhr.ca/en/wp-content/uploads/2015/08/Buried_Voices.pdf. Accessed 19 Feb 2016.

[54] Vividata. Newspaper topline readership: Vividata 205 Q2. 2015. http://vividata.ca/wp-content/uploads/2015/10/TOPLINE-Readership-by-Newspaper-Vividata-2015-Q21.pdf. Accessed 3 Jan 2016.

[55] Landis JR, Koch GG (1977). The measurement of observer agreement for categorical data. Biometrics.

[56] Fereday J, Muir-Cochrane E (2006). Demonstrating rigor using thematic analysis: a hybrid approach of inductive and deductive coding and theme development. Int J Qual Methods.

[57] Braun V, Clarke V (2006). Using thematic analysis in psychology using thematic analysis in psychology. Qual Res Psychol.

[58] de Vreese C, Boomgaarden H (2003). Valenced news frames and public support for the EU: linking content analysis and experimental data. Eur J Commun.

[59] Iyengar S (1991). Is anyone responsible?.

[60] Joffe H, Yardley L, Marks DF, Yardley L (2003). Content and thematic analysis. Research methods for clinical and health Pyschology.

[61] Fitzgerald M (2008). First Nations drinking-water plan gets $330M; “Big-Picture View.” Don Mills.

[62] Ashawasegai J (2012). Nations wary about plans to reopen mine.

[63] Fontaine P (2007). Budget a blow to First Nations’ hopes: First Nations rejected by Budget 2007.

[64] Ha TT (2000). Canada violates native rights, UN told.

[65] Diebel L (2010). Native community lacks safe water.

[66] Howlett K (2005). Ontario orders survey of water on reserves.

[67] MacKinnon M (2001). Vital to improve water quality on reserves, group says.

[68] Martin D (2006). Time for action, Prentice says.

[69] Steele D (2010). Page 5 Chatter.

[70] Fife R (2001). Native chiefs welcome PM’s intervention.

[71] Welsh J (2010). Disturbing double standard.

[72] Taillon J (2002). A Walkerton waiting to happen.

[73] Anonymous (2015). Unsafe to drink? Hard to swallow.

[74] Brean J (2015). Trudeau’s to-do list has a lot of urgent items.

[75] Health Canada. Drinking water and wastewater. 2014. http://www.hc-sc.gc.ca/fniah-spnia/promotion/public-publique/water-eau-eng.php. Accessed 26 Sep 2014.

[76] Furgal CM, Garvin TD, Jardine CG (2010). Trends in the study of aboriginal health risks in Canada. Int J Circumpolar Health.

[77] Furgal C, Seguin J (2006). Climate change, health, and vulnerability in Canadian northern Aboriginal communities. Environ Health Perspect.

[78] Hoover E, Cook K, Plain R, Sanchez K, Waghiyi V, Miller P (2015). Indigenous peoples of North America: Environmental Exposures and Reproductive Justice. Environ Health Perspect.

[79] Palmater PD (2011). Stretched beyond human limits: death by poverty in First Nations. Can Rev Soc Policy..

[80] Eggertson L (2007). Physicians challenge Canada to make children, youth a priority. Can Med Assoc J.

[81] Barclay K, Liu JH (2003). Who gets voice? (Re)presentation of bicultural relations in New Zealand print media. NZ J Psychol.

[82] Walters D, Spence N, Kuikman K, Singh B (2012). Multi-barrier protection of drinking water systems in Ontario: a comparison of First Nation and non-First Nation communities. Int Indig Policy J.

[83] Barnes MD, Hanson CL, Novilla LMB, Meacham AT, McIntyre E, Erickson BC (2008). Analysis of media agenda setting during and after Hurricane Katrina: Implications for emergency preparedness, disaster response, and disaster policy. Am J Public Health.

[84] Carvalho A, Burgess J (2005). Cultural circuits of climate change in U.K. broadsheet newspapers, 1985-2003. Risk Anal.

[85] Anderson A (1997). Media, culture and the environment.

[86] National Post. Flooding forces full evacuation of Kashechewan; more than 1,800 displaced from remote James Bay community. 2015. http://news.nationalpost.com/news/canada/flooding-forces-full-evacuation-of-kashechewan-more-than-1800-displaced-from-remote-james-bay-community. Accessed 25 Jan 2017.

[87] Hurlimann A, Dolnicar S (2012). Newspaper coverage of water issues in Australia. Water Res.

[88] McCullough J, Farahbakhsh K (2012). Square peg, round hole: First Nations drinking water infrastructure and federal policies, programs, and processes. Int Indig Policy J.

[89] White JP, Murphy L, Spence N (2012). Water and peoples: Canada’s paradox. Int Indig Policy J.

[90] AANDC (Aboriginal Affairs and Northern Development Canada): First Nations water and wastewater action plan progress report April 2009 – March 2010. 2010. https://www.aadnc-aandc.gc.ca/eng/1100100034932/1100100034943. Accessed 26 Nov 2014.

[91] Indigenous and Northern Affairs Canada: Backgrounder - Safe Drinking Water for First Nations Act. 2013. http://www.aadnc-aandc.gc.ca/eng/1330529331921/1330529392602. Accessed 8 Jan 2016.

[92] OAGC (Office of the Auditor General of Canada): Report of the commissioner of the environment and sustainable development to the House of Commons: Chapter 5 drinking water in First Nations communities. 2005. http://www.oag-bvg.gc.ca/internet/docs/c20050905ce.pdf. Accessed 21 Feb 2015.

[93] Simeone T: Safe drinking water in First Nations communities. 2010. http://www.parl.gc.ca/content/LOP/ResearchPublications/prb0843-e.pdf. Accessed 26 Nov 2014.

[94] Hrudey SE, Payment P, Huck PM, Gillham RW, Hrudey EJ (2003). A fatal waterborne disease epidemic in Walkerton, Ontario: Comparison with other waterborne outbreaks in the developed world. Water Sci Technol.

[95] Holme R (2003). Drinking water contamination in Walkerton, Ontario: Positive resolutions from a tragic event. Water Sci Technol.

[96] McGregor D (2012). Traditional knowledge: considerations for protecting water in Ontario. Int Indig Policy J..

[97] AANDC (Aboriginal Affairs and Northern Development Canada): Safe drinking water for First Nations Act. 2014. https://www.aadnc-aandc.gc.ca/eng/1330528512623/1330528554327. Accessed 26 Nov 2014.

[98] Dotson DM, Jacobson SK, Kaid LL, Carlton JS (2012). Media coverage of climate change in Chile: a content analysis of conservative and liberal newspapers. Environ Commun..

[99] Boykoff MT, Boykoff JM (2007). Climate change and journalistic norms: a case-study of US mass-media coverage. Geoforum.

[100] Young N (2011). Representations of climate change in canadian national print media: the banalization of global warming. Can Rev Sociol..

[101] Boykoff MT, Roberts JT: Media Coverage of Climate Change: Current Trends, Strengths, Weaknesses. http://hdr.undp.org/fr/rapports/mondial/rmdh2007-2008/documents/Boykoff, Maxwell and Roberts, J. Timmons.pdf (2007). Accessed 3 Jan 2016.

[102] Charron D, Thomas MK, Waltner-Toews D, Aramini JJ, Edge T, Kent RA (2004). Vulnerability of waterborne diseases to climate change in Canada: a review. J. Toxicol. Environ. Heal. Part A..

[103] Statistics Canada. Appendix D: Mother tongue and home language: classifications from 2011, 2006 and 2001. 2011. http://www12.statcan.gc.ca/census-recensement/2011/ref/dict/app-ann004-eng.cfm. Accessed 26 Nov 2014.

