# Supplementary material for: How does the media portray drinking water security in Indigenous communities in Canada? An analysis of Canadian newspaper coverage from 2000-2015
Source: BMC Public Health. 2017 Mar 27;17:282. doi: 10.1186/s12889-017-4164-4 (PMC5368908; doi:10.1186/s12889-017-4164-4)
Supplement: Additional file 1: — Descriptive information, drinking water issues, and framing of articles extracted from articles for deductive qualitative analysis. (DOCX 15 kb) [file 12889_2017_4164_MOESM1_ESM.docx]

**Additional table 1.** Descriptive information, drinking water issues, and framing of articles extracted from articles for deductive qualitative analysis.

| Coding categories | Action | Options |
| --- | --- | --- |
| Descriptive information | | |
| Name of newspaper | Check one | *Toronto Star The Globe and Mail National Post Windspeaker* |
| Date | Input date | DD/MM/YY |
| Page number | Input number | # |
| Type of article | Check one | News Editorial Opinion or Column Letter to the editor Not available |
| Author | Check one | Journalist Politician Public Anonymous |
| Word count | Check one | 1-250 251-500 501-1000 1001-2000 2000+ |
| Sources quoted | Check all that apply | Politician Government Indigenous person  Elected Indigenous  Non-elected Indigenous Academic or scientist Report  Other |
| Scientific evidence provided (e.g., reference to a report) | Check one | Yes No |
| Drinking water issues | | |
| Issues | Check all that apply | Blame Government responses Drinking water infrastructure Regulatory framework Drinking water policy Source water protection / multi-barrier approach Governance  Climate change / global warming |
| Community | Check all that apply | Aboriginal / Indigenous First Nations Métis Inuit |
| Frame | | |
| Frame type | Check one | Episodic Thematic |
| Focus | Check one | Drinking water issue Other issue |
| Valence / tone | Check one | Positive Negative Neutral |
| Content | Check one | Substantive Ambiguous |
